# CD74 Promotes Cyst Growth and Renal Fibrosis in Autosomal Dominant Polycystic Kidney Disease

**DOI:** 10.3390/cells13060489

**Published:** 2024-03-11

**Authors:** Julie Xia Zhou, Alice Shasha Cheng, Li Chen, Linda Xiaoyan Li, Ewud Agborbesong, Vicente E. Torres, Peter C. Harris, Xiaogang Li

**Affiliations:** 1 Department of Internal Medicine, Mayo Clinic, Rochester, MN 55905, USA; 2 Department of Biochemistry and Molecular Biology, Mayo Clinic, Rochester, MN 55905, USA

**Keywords:** CD74, cystogenesis, fibrosis, ADPKD

## Abstract

The progression of autosomal dominant polycystic kidney disease (ADPKD), an inherited kidney disease, is associated with renal interstitial inflammation and fibrosis. CD74 has been known not only as a receptor of macrophage migration inhibitory factor (MIF) it can also have MIF independent functions. In this study, we report unknown roles and function of CD74 in ADPKD. We show that knockout of CD74 delays cyst growth in *Pkd1* mutant kidneys. Knockout and knockdown of CD74 (1) normalize PKD associated signaling pathways, including ERK, mTOR and Rb to decrease *Pkd1* mutant renal epithelial cell proliferation, (2) decrease the activation of NF-κB and the expression of MCP-1 and TNF-alpha (TNF-α) which decreases the recruitment of macrophages in *Pkd1* mutant kidneys, and (3) decrease renal fibrosis in *Pkd1* mutant kidneys. We show for the first time that CD74 functions as a transcriptional factor to regulate the expression of fibrotic markers, including collagen I (Col I), fibronectin, and α-smooth muscle actin (α-SMA), through binding on their promoters. Interestingly, CD74 also regulates the transcription of MIF to form a positive feedback loop in that MIF binds with its receptor CD74 to regulate the activity of intracellular signaling pathways and CD74 increases the expression of MIF in ADPKD kidneys during cyst progression. We further show that knockout of MIF and targeting MIF with its inhibitor ISO-1 not only delay cyst growth but also ameliorate renal fibrosis through blocking the activation of renal fibroblasts and CD74 mediated the activation of TGF-β-Smad3 signaling, supporting the idea that CD74 is a key and novel upstream regulator of cyst growth and interstitial fibrosis. Thus, targeting MIF-CD74 axis is a novel therapeutic strategy for ADPKD treatment.

## 1. Introduction

Autosomal dominant polycystic kidney disease (ADPKD) is characterized by progressive renal cyst formation and expansion, which is associated with renal interstitial inflammation and fibrosis and ultimately leads to end stage kidney disease (ESKD). ADPKD is an inherited disease that is caused by mutations in PKD1 and PKD2 genes, encoding polycystin-1 (PC1) and polycystin-2 (PC2), respectively [1]. The cystic renal epithelial cell proliferation and apoptosis are regulated by multiple dysregulated signaling pathways in ADPKD that are identified to be potential therapeutic targets in animal models of PKD [2,3,4]. Interstitial inflammation has been reported in human and animal models of PKD, which aggravates PKD mediated by TNF-α signaling [5] and macrophages recruitment [6,7]. Renal interstitial fibrosis is one of the major pathological changes in ADPKD, whereas the underlying mechanisms remain elusive.

CD74 is a type-II transmembrane glycoprotein, which has been recognized as an important regulator of immunity and inflammation. In addition to being a cell membrane receptor of macrophage migration inhibitory factor (MIF), CD74 by itself is a multifunctional protein that can regulate intracellular trafficking and function as a chaperone to modulate B, T, and dendritic cell responses [8,9]. CD74 also regulates the functions of non-immune and non-tumor cells [8,10]. Elevated CD74 expression is found in renal tubular cells in some forms of chronic kidney disease, and in glomerular podocytes and parietal cells in human metabolic nephropathies, experimental diabetic nephropathy, glomerulonephritis, as well as in human clear cell renal cell carcinoma [11,12,13,14]. Overexpression of CD74 has been reported to increase human embryonic kidney cell proliferation and the activation of NF-κB [14], which results in the upregulation of NF-κB-dependent genes, including those encoding cytokines [15]. Activation of NF-κB has been reported in cystic renal epithelial cells [4]. However, the role and mechanisms of CD74 in regulation of cyst growth and renal fibrosis in ADPKD remain unknown.

In this study, we investigate the molecular mechanisms that link CD74 with cyst growth and renal inflammation and fibrosis in ADPKD and address a novel role of CD74 as transcriptional factor to regulate the transcription of MIF and fibrotic markers, which indicates a positive feedback loop between MIF and CD74. This study leads to a better understanding of the pathological mechanisms of renal cyst formation and has a direct potential to accelerate the development of potential therapies.

## 2. Methods

### 2.1. Cell Cultures and Reagents

Pkd1 homozygous null PN24 cells, provided by Stefan Somlo through the George M. O’Brien Kidney Center at Yale University (New Haven, CT, USA), were cultured as described [16]. Murine IMCD3 cells were maintained at 37 °C in 5% CO_2_ in DMEM (Invitrogen, Waltham, MA, USA) supplemented with 10% FBS. Normal rat kidney 49F (NRK-49F) cells were cultured as described [17]. Recombinant TGF-β and MIF were purchased from R&D Systems (Minneapolis, MN, USA).

### 2.2. Western Blot Analysis

Cell pellets were lysed at 4 °C for 30 min with modified lysis buffer consisting of 20 mM Tris-HCl (pH 7.5), 1% Triton X-100, 150 mM NaCl, 1% glycerol, 0.5 mM dithiothreitol, and 1 mM sodium vanadate plus protease inhibitor (Roche Applied Science, Penzberg, Germany). Cell extracts were clarified by centrifuging at 16,000× *g* for 15 min at 4 °C, and the supernatants were collected for Western blot analysis. The proteins were analyzed by blotting with the following primary antibodies against: α-SMA (1:2000, A2547, Sigma-Aldrich, St. Louis, MO, USA), fibronectin (1:1000, #63779, Cell Signaling Technology, Danvers, MA, USA), Smad3 (1:1000, #9523, Cell Signaling Technology, Danvers, MA, USA), phospho-Smad3 (1:1000, #9520, Cell Signaling Technology), phospho-Rb (1:1000, #9307, Cell Signaling Technology), Rb (1:100, sc-73598, Santa Cruz Biotechnology, Dallas, TX, USA), AKT (1:1000, #9272, Cell Signaling Technology, Danvers, MA, USA), phospho-Akt (1:1000, #9271, Cell Signaling Technology), S6 (1:1000, #2217, Cell Signaling Technology), phospho-S6 (1:1000, #2211, Cell Signaling Technology), MIF (1:100, SC-20121, Santa Cruz Biotechnology, Dallas, TX, USA), CD74 (1:100, SC-20082, Santa Cruz Biotechnology) anti-ERK (#9107, Cell Signaling Technology), phospho-ERK (#9101, Cell Signaling Technology), and β-actin (1:1000, A5316, Sigma-Aldrich). The secondary antibodies including donkey anti-rabbit IgG–horseradish peroxidase, donkey anti-mouse IgG–horseradish peroxidase, and donkey anti-goat IgG–horseradish peroxidase (Santa Cruz Biotechnology; 1:8000 dilution) were used.

### 2.3. Immunohistochemistry and Immunofluorescence Staining

Kidneys were fixed with 4% paraformaldehyde (pH 7.4). Paraffin-embedded sections (5 μm) were subjected to the staining. After antigen retrieval, tissue sections were incubated with anti-F4/80 primary antibody (1:100, MCA497, BIO-RAD, Hercules, CA, USA), followed by incubation with Fluro-488 anti-rat IgG secondary antibody. Then the tissue sections were mounted in Prolong Gold Anti-fade reagent with DAPI (Invitrogen, Waltham, MA, USA). A rabbit anti-Ki67 antibody (1:100, ab15580; Abcam, Cambridge, UK) and a Fluro-488 anti-rabbit IgG secondary antibody were used for Ki67 staining. A goat anti-α-SMA antibody (1:100, Santa Cruz Biotechnology), a biotinylated secondary antibody (1:100, Santa Cruz Biotechnology), and the diaminobenzidine (DAB) substrate system were used for α-SMA staining. Kidney sections were counterstained by hematoxylin. Collagen fibers in kidney sections were stained with Masson trichrome and picrosirus red. Images were analyzed using a NIKON ECLIPSE 80i microscope.

NRK-49F cells were treated with either TGF-β (10 ng/mL) or vehicle for 24 h. Then the cells were fixed with 4% paraformaldehyde, followed by permeabilization with 0.1% Triton X-100. After blocking with 2% BSA, cells were incubated with anti-CD74 antibody (1:100 dilution, Santa Cruz Biotechnology). A Fluro-488 anti-mouse IgG secondary antibody and prolong gold anti-fade reagent with DAPI (Invitrogen) were used. Images were captured with a Nikon AX-R confocal microscope (Minato City, Japan).

### 2.4. TUNEL Assay

TUNEL assay for kidney sections was performed according to the manufacturer’s protocols (In Situ Death Detection Kit; Roche, Basel, Switzerland). Prolong Gold Anti-fade reagent with DAPI (Invitrogen) was used. Immunofluorescence images were obtained with a NIKON ECLIPSE 80i microscope.

### 2.5. Cystic Index

The cyst area and total kidney area were measured by Image J from sagittal sections of whole kidneys. Cystic index is the total area of cysts within the total area of the kidney. Cystic index = (total cystic area/total kidney area) × 100.

### 2.6. Quantitative Reverse-Transcription PCR (qRT-PCR)

Total RNA (1 μg) extracted by the RNeasy plus mini kit (Qiagen, Hilden, Germany) was used for reverse transcription reactions to synthesize cDNA using Iscript cDNA Synthesis Kit (BIO-RAD. The RNA expression was analyzed by real-time PCR using iTaq SYBER Green Supermix with ROX (Bio-Rad) in a CFX Connect Real-time PCR detection system. The following program of thermal cycling was used: 40 cycles of 10 s at 95 °C and 20 s at 61 °C. A melting curve was run after each PCR cycle, followed by a cooling step. Triplicate samples were run in each experiment. The relative gene expression levels of target genes were analyzed by the 2^−ΔΔ^^C^_T_ method and using reference gene actin or GAPDH. Mouse genes were amplified using the following primers:

MIF-F, 5′-AGAACCGCAACTACAGTAAGC-3′; MIF-R, 5′-ACTCAAGCGAAGGTGGAAC-3′;

TGF-β-F, 5′-CCTGAGTGGCTGTCTTTTGA-3′; TGF-β-R, 5′-CGTGGAGTTTGTTATCTTTGCTG3′;

TNF-α-F, 5′-CTTCTGTCTACTGAACTTCGGG-3′; TNF-α-R, 5′-CAGGCTTGTCACTCGAATTTTG-3′;

MCP-1-F, 5′-GTCCCTGTCATGCTTCTGG-3′; MCP-1-R, 5′-GCTCTCCAGCCTACTCATTG-3′;

ColI-F 5′-CGTAAGCACTGGTGGACAGA-3′; ColI-R 5′-TCTGAGGAATGCCAGCTGCA-3′;

ColIII-F 5′-GAAGTCTCTGAAGCTGATGGG-3′; ColIII-R 5′-TTGCCTTGCGTGTTTGATATTC-3′;

Fibronectin-F 5′- AGACCATACCTGCCGAATGTA G-3′; Fibronectin-R 5′-GAGAGCTTCCTGTCCTGT AGAG-3′; Actin-F, 5′-AAGAGCTATGAGCTGCCTGA-3′; Actin-R, 5′-TACGGATGTCAACGTCACAC-3′;

Rat genes were amplified using the following primers:

ColI-F 5′-ATCAGCCCAAACCCCAAGGAG A-3′; ColI-R 5′-CGCAGGAAGGTCAGCTGGATA G-3′;

ColIII-F 5′-TGATGGGATCCAATGAGGGAGA-3′; ColIII-R 5′-GAGTCTCATGGCCTTGCGTGTTT-3′;

TGF-β-F 5′-CCCCTGGAAAGGGCTCAACAC-3′; TGF-β-R, 5′-TCCAACCCAGGTCCTTCCTAAAGTC-3′.

Fibronectin-F 5′-CATGGCTTTAGGCGAACCA-3′; Fibronectin-R 5′-CATCTACATTCGGCAGGTATGG-3′; α-SMA-F 5′-GATCACCATCGGGAATGAACG C-3′; α-SMA-R 5′-CTTAGAAGCATTTGCGGTGGAC-3′; GAPDH-F 5′-CCATTCTTCCACCTTTGATGCT-3′; GAPDH-R 5′-TGTTGCTGTAGCCATATTCAT TGT-3′.

### 2.7. MTS Assay

PN24 cells were transfected with control siRNA or CD74 siRNA for 48 h. NRK-49F cells were treated with 0, 12.5, or 25 ng/mL MIF for 48 h. Cell viability was measured by using CellTiter 96 AQ_ueous_ One Solution Cell proliferation Assay (MTS) kit (Promega, Madison, WI, USA), according to the manufacturer’s instructions. Briefly, 20 μL of CellTiter 96 AQ_ueous_ One Solution Regent were pipetted into each well of the 96-well assay plate. After incubation of the plate at 37 °C for 4 h, the absorbance at 490 nm were recorded by a 96-well plate reader.

### 2.8. RNA Interference

Cells were transfected with RNA oligonucleotides targeting mouse CD74, rat CD74, and control siRNA (Dharmacon, St. Louis, MO, USA) using DharmaFECT siRNA transfection reagent (Dharmacon) for 48 h. Subsequentlythey were harvested for Western blot analysis and RT-qPCR.

### 2.9. Chromatin Immunoprecipiation Assay

The ChIP assay was performed according to the protocol previously described [18]. Chromatin DNA was subjected to immunoprecipitation (IP) with anti-CD74 or normal rabbit IgG and then washed, after which the DNA-protein cross-links were reversed. The recovered DNA was analyzed by qPCR for the binding of Col I, Col III, α-SMA, fibronectin, MIF, and TGF-β promoters by using the following PCR primers: Col I promoter forward 5′-GGCTGGAGAAAGGTGGGTCT-3′; Col I promoter reverse 5′-CCCAGGTATGCAGGGTAGGA-3′; α-SMA promoter forward 5′-CATGCACGTGGACTGTACCT-3′; α-SMA promoter reverse 5′-AAAGATGCTTGGGTCACCTG-3′; fibronectin promoter forward 5′-CGTACCCTGGAAAGTC-3′; fibronectin promoter reverse 5′-CTAAGCCTACCTAACACCGA-3′; MIF promoter forward 5′-CAGGATCTAATGTGAGCAGGG-3′; MIF promoter reverse 5′-GAGGATGCTTGGAGAACTCTG-3′.

### 2.10. Mouse Strains and Treatment

All animal protocols were approved and conducted in accordance with the Laboratory Animal Resources of University of Kansas Medical Center and Institutional Animal Care and Use Committee regulations (Protocol #2015-2092).

*Pkd1^flox/flox^* mice (B6; 129S4-*Pkd1*^tm2Ggg^/J; stock 010671; Jackson Laboratories, (Bar Harbar, ME, USA) possess loxP sites on either side of exons 2–4 of *Pkd1* [19]. *Ksp-Cre* mice express Cre recombinase under the control of the Ksp-cadherin promoter [20]. *Pkhd1-Cre* transgenic mice express Cre recombinase under the control of the Pkhd1 promoter [21]. *CD74^−/−^* mice (B6.129S-*Cd74^tm1Liz^*/J; stock 002729; Jackson Laboratories) possess a MC1neopA cassette that replaces the first intron and 11 nucleotides of the second exon.

*Pkd1^flox/+^*:*Ksp-Cre* mice were generated by cross-breeding *Pkd1^flox/flox^* female mice with *Ksp-Cre* male mice. *Pkd1^flox/+^*:*CD74^+/−^*:*Ksp-Cre* mice were generated by cross-breeding *Pkd1^flox/+^*:*Ksp-Cre* female mice with *CD74^−/−^* male mice. *Pkd1^flox/flox^*:*CD74^+/+^*:*Ksp-Cre* mice and *Pkd1^flox/flox^*:*CD74^−/−^*:*Ksp-Cre* mice were generated by cross-breeding *Pkd1^flox/+^*:*CD74^+/−^*:*Ksp-Cre* female and male mice. *Pkd1^flox/flox^*:*CD74^+/+^*:*Ksp-Cre* mice and *Pkd1^flox/flox^*:*CD74^−/−^*:*Ksp-Cre* mice were sacrificed at postnatal day 7. The kidneys and serum were harvested for further analysis.

*Pkd1^flox/+^*:*Pkhd1-Cre* mice were generated by cross-breeding *Pkd1^flox/flox^* female mice with *Pkhd1-Cre* male mice. *Pkd1^flox/+^*:*CD74^+/−^*:*Pkhd1-Cre* mice were generated by cross-breeding *Pkd1^flox/+^*:*Pkhd1-Cre* female mice with *CD74^−/−^* male mice. *Pkd1^flox/flox^*:*CD74^+/+^*:*Pkdh1-Cre* mice and *Pkd1^flox/flox^*:*CD74^−/−^*:*Pkhd1-Cre* mice were generated by cross-breeding *Pkd1^flox/+^*:*CD74^+/−^*:*Ksp-Cre* female and male mice. *Pkd1^flox/flox^*:*CD74^+/+^*:*Pkhd1-Cre* mice and *Pkd1^flox/flox^*:*CD74^−/−^*:*Pkhd1-Cre* mice were sacrificed at postnatal day 21. The kidneys and serum were harvested for further analysis.

The Pkd1 hypomorphic *Pkd1^nl/nl^* mice and MIF knockout MIF^−/−^ mice were generated as previously described [16]. *Pkd1^nl/+^*:*MIF^+/−^* mice were generated by cross-breeding *Pkd1^nl/nl^* female mice with MIF^−/−^ male mice. *Pkd1^nl/nl^*:*MIF^+/+^* mice and *Pkd1^nl/nl^*:*MIF^−/−^* mice were generated by cross-breeding *Pkd1^nl/nl^* female mice with MIF^−/−^ male mice. *Pkd1^nl/nl^*:*MIF^+/+^* mice and *Pkd1^nl/nl^*:*MIF^−/−^* mice were sacrificed at postnatal day 28. The kidneys and serum were harvested for further analysis. Kidney tissues from postnatal day 28 *Pkd1^nl/nl^* mice treated with DMSO or ISO were generated from previous study [16].

### 2.11. Statistics

All data are presented as the mean ± SEM and analyzed in GraphPad Prism version 10 to determine the significance of the differences. The datasets with sample size of *n* = 3 were analyzed by using a nonparametric test, the Mann-Whitney U test. The unpaired student’s *t*-test assuming unequal variance (Welch’s *t*-test) was used for the analysis of the datasets with sample size *n* > 3. The datasets with multiple groups were analyzed by one-way ANOVA (one independent variable, multiple groups). A *p* value that is less than or equal to 0.05 is considered statistically significant.

## 3. Results

### 3.1. Double Knockout of CD74 and Pkd1 Delays Cyst Growth in Two Pkd1 Mutant Mouse Models

Our previous report showed that the mRNA and protein expression of CD74 were upregulated in *Pkd1* mutant renal epithelial cells, including *Pkd1* null mouse embryonic kidney (MEK) cells and postnatal *Pkd1* homozygous PN24 cells, and its expression was also increased in cyst lining epithelial cells in human ADPKD kidneys compared with normal kidneys [16].

**Figure 1:**
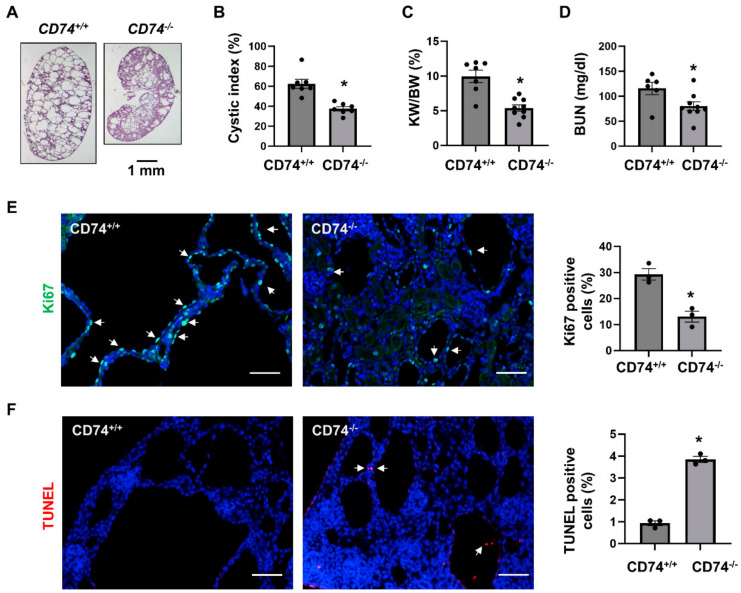
Knockout of CD74 delays cyst growth in *Pkd1^flox/flox^:Ksp-Cre* mice. (**A**) Histologic examination of PN7 kidneys from *Pkd1^flox/flox^:Ksp-Cre*:*CD74^+/+^* (*CD74^+/+^*) mice (*n* = 9) and *Pkd1^flox/flox^*:*Ksp-Cre*:*CD74^−/−^* (*CD74^−/−^*) mice (*n* = 7). Scale bar: 1 mm. (**B**) Cystic index was significantly decreased in PN7 kidneys from *CD74^−/−^* mice compared to *CD74^+/+^* mice. Mean ± SEM of all sections quantified for each condition are shown. *p* < 0.01. (**C**,**D**) KW/BW ratios (**C**) and BUN levels (**D**) of PN7 *CD74^+/+^* and *CD74^−/−^* mice. Mean ± SEM are shown * *p* ≤ 0.05 by student’s *t*-test. (**E**) Cell proliferation was decreased in kidneys of *CD74^−/−^* mice (*n* = 3) compared with that of *CD74^+/+^* mice (*n* = 3), as detected by Ki67 staining. Scale bar: 50 µm. Arrows indicate the Ki67 positive cells. Green: Ki67. Blue: DAPI. Mean ± SEM are shown. * *p* ≤ 0.05 by Mann-Whitney U test. (**F**) Cell apoptosis was increased in kidneys of *CD74^−/−^* mice compared with those of *CD74^+/+^* mice, as detected by TUNEL assay. Scale bar: 50 µm. Red: TUNEL positive, Blue: DAPI. Mean ± SEM are shown. * *p* ≤ 0.05 by Mann-Whitney U test.

In order to explore the function of CD74 in vivo, we generated *Pkd1^flox/flox^*:*Ksp-Cre*:*CD74^−/−^* mice. We found that knockout of CD74 delayed cyst growth in kidneys from *Pkd1^flox/flox^*:*Ksp-Cre*:*CD74^−/−^* mice (*n* = 9) at postnatal day 7 (PN7) compared to that in age matched *Pkd1^flox/flox^*:*Ksp-Cre*:*CD74^+/+^* mice (*n* = 7) (Figure 1A). The knockout efficiency of CD74 was confirmed by Western blot (Supplemental Figure S1). Knockout of CD74 decreased cyst index (Figure 1B), kidney weight to body weight ratios (KW/BW) (Figure 1C) and blood urea nitrogen (BUN) (Figure 1D) in *Pkd1^flox/flox^*:*Ksp-Cre*:*CD74^−/−^* mice compared to those from *Pkd1^flox/flox^*:*Ksp-Cre*:*CD74^+/+^* mice. In addition, the cyst lining epithelial cell proliferation as examined with Ki67 staining was decreased (Figure 1E) and the cystic lining epithelial cell apoptosis as examined with TUNEL assay was increased (Figure 1F) in *Pkd1* and CD74 double knockout kidneys compared to that in *Pkd1* conditional knockout but CD74 wild type kidneys.

**Figure 2:**
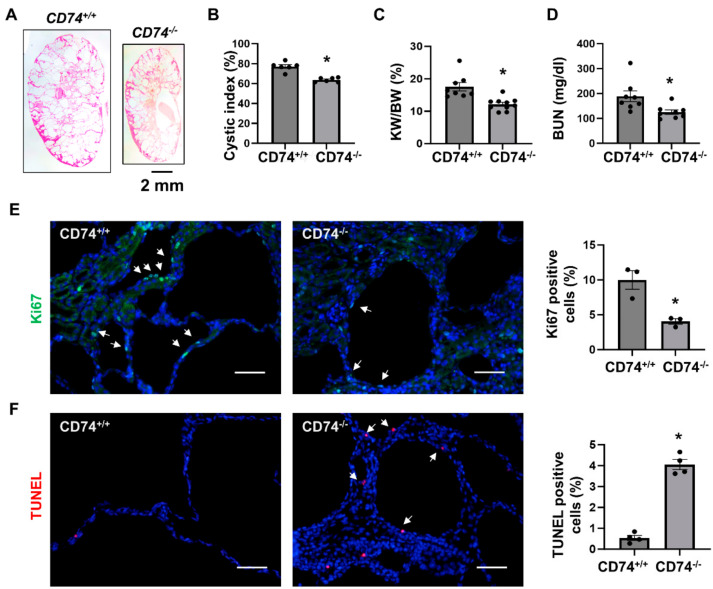
Knockout of CD74 delays cyst growth in *Pkd1^flox/flox^*:*Pkhd1-Cre* mice. (**A**) Histologic examination of PN21 kidneys from *Pkd1^flox/flox^*:*Pkhd1-Cre*:*CD74^+/+^* (*CD74^+/+^*) mice (*n* = 8) and *Pkd1^flox/flox^*:*Pkhd1-Cre*:*CD74^−/−^* (*CD74^−/−^*) mice (*n* =10). Scale bar: 2 mm. (**B**) Cystic index was significantly decreased in PN21 kidneys from *CD74^−/−^* mice compared to *CD74^+/+^* mice. Mean ± SEM of all sections quantified for each condition are shown. *p* < 0.01 by student’s *t*-test. (**C**,**D**) KW/BW ratios (**C**) and BUN levels (**D**) of PN21 *CD74^+/+^* and *CD74^−/−^* mice. Mean ± SEM are shown. *p* < 0.01 or < 0.05 by student’s *t*-test. (**E**) Cell proliferation was decreased in kidneys of *CD74^−/−^* mice (*n* = 3) compared with that of *CD74^+/+^* mice (*n* = 3), as detected by Ki67 staining. Scale bar: 50 µm. Arrows indicate the Ki67 positive cells. Green: Ki67. Blue: DAPI. Mean ± SEM are shown. * *p* ≤ 0.05 by Mann-Whitney U test. (**F**) Cell apoptosis was increased in kidneys of *CD74^−/−^* mice (*n* = 4) compared with that of *CD74^+/+^* mice (*n* = 4), as detected by TUNEL assay. Scale bar: 50 µm. Red: TUNEL positive, Blue: DAPI. Mean ± SEM are shown. *p* < 0.01 by student’s *t*-test.

To further confirm the in vivo function of CD74 in another *Pkd1* knockout mouse model, we generated *Pkd1^flox/flox^*:*Pkhd1-Cre*:*CD74^−/−^* mouse. Consistent with the findings in *Pkd1^flox/flox^*:*Ksp-Cre* mice, we found that knockout of CD74 also delayed cyst growth in postnatal day 21 (PN21) *Pkd1^flox/flox^*:*Pkhd1-Cre*:*CD74^−/−^* kidneys (*n* = 8) compared to that in age matched *Pkd1^flox/flox^*:*Pkhd1-Cre:CD74^+/+^* kidneys (*n* = 10) (Figure 2A) characterized by the decrease of cystic index, KW/BW ratios and BUN levels (Figure 2B–D) as well as cyst lining epithelial cell proliferation as examined by Ki67 staining (Figure 2E), but induced cyst lining epithelial cell apoptosis as examined by TUNEL assay (Figure 2F). These results indicated that upregulation of CD74 promotes renal cyst growth by regulating cystic renal epithelial cell proliferation and apoptosis in *Pkd1* mutant mouse kidneys.

### 3.2. CD74 Promotes Pkd1 Mutant Renal Epithelial Cell Proliferation through the Activation of MAPK, mTOR and NF-κB Pathways

**Figure 3:**
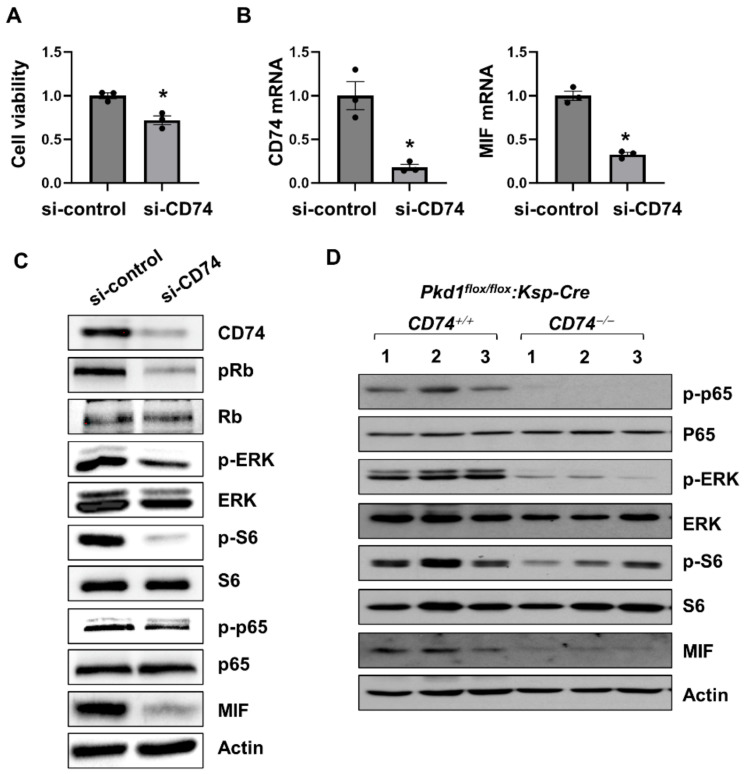
CD74 promotes *Pkd1* mutant renal epithelial cell proliferation through the activation of MAPK, mTOR and NF-κB pathways. (**A**) The cell viability of *Pkd1* null cells treated with control siRNA and CD74 siRNA by MTS assay. * *p* ≤ 0.05 by Mann-Whitney U test. (**B**) The mRNA of CD74 and MIF in *Pkd1* null cells treated with control siRNA and CD74 siRNA by qRT-PCR. * *p* ≤ 0.05 by Mann-Whitney U test. (**C**) Western blot analysis of the expression of phospho-Rb, Rb, phospho-ERK, ERK, phospho-p65, p65, phospho-S6, S6, and MIF in *Pkd1* null cells treated with control siRNA and CD74 siRNA. (**D**) Western blot analysis of the expression of phospho-p65, p65, phospho-ERK, ERK, phospho-S6, S6, MIF, and CD74 in kidneys from three different *Pkd1^flox/flox^*:*Ksp-Cre*:*CD74^+/+^* mice and *Pkd1^flox/flox^*:*Ksp-Cre*:*CD74^−/−^* mice.

To determine a direct effect of CD74 on renal cell proliferation and its associated signaling pathways, first, we found that knockdown of CD74 decreased *Pkd1* mutant renal epithelial cell proliferation as examined with MTS assay (Figure 3A). The knockdown efficiency of CD74 with siRNA was confirmed by qRT-PCR analysis (Figure 3B). Knockdown of CD74 also decreased the phosphorylation of ERK, S6, Rb, and p65, but not the expression of their total proteins (Figure 3C) in *Pkd1* mutant renal epithelial cells. In addition, knockout of CD74 also decreased the activation and phosphorylation of ERK, S6, and p65 in *Pkd1* conditional knockout kidneys (Figure 3D). These results suggest that CD74 regulates *Pkd1* mutant renal epithelial cell proliferation through PKD associated signaling pathways. The downregulation of MIF mRNA and protein (Figure 3B–D) in CD74 knockdown cells and knockout kidneys is unexpected, suggesting that there is a positive feedback loop between MIF and CD74.

### 3.3. Deletion of CD74 Reduces the Recruitment of Macrophages and the Expression of Inflammatory Factors in Pkd1^flox/flox^:Ksp-Cre and Pkd1^flox/flox^:Pkhd1-Cre Kidneys

**Figure 4:**
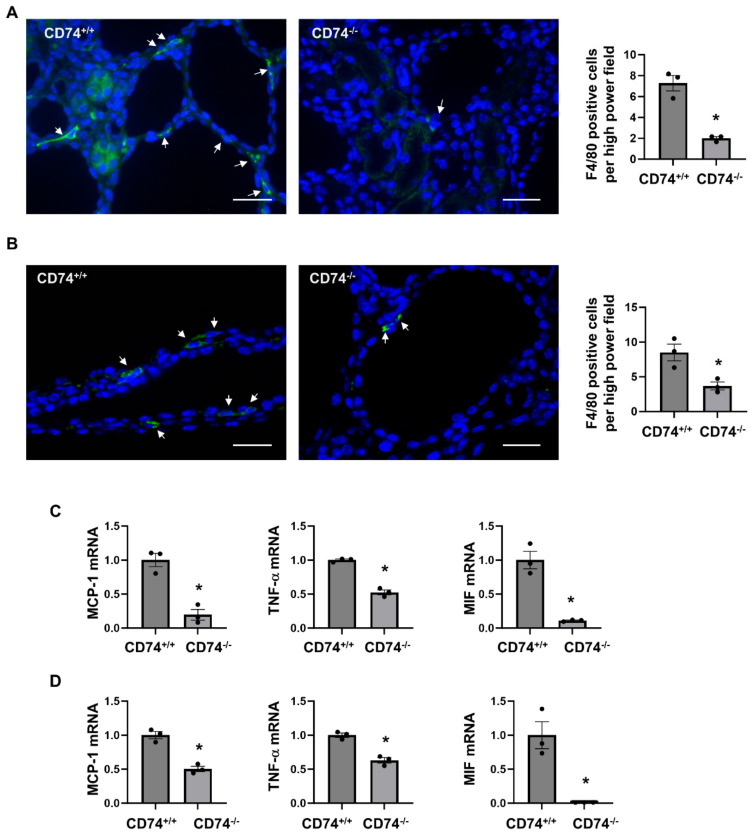
Knockout of CD74 decreases macrophage recruitment and expression of inflammatory factors in cystic kidneys. (**A**) Immunofluorescent staining of macrophages by anti-F4/80 antibody in cystic kidneys of *Pkd1^flox/flox^*:*Ksp-Cre*:*CD74^+/+^* mice and *Pkd1^flox/flox^*:*Ksp-Cre*:*CD74^−/−^* mice. The average numbers of F4/80 positive cells per high power field image were present. Arrows indicate the F4/80 positive cells. Green: F4/80. Blue: DAPI. *n* = 3. * *p* ≤ 0.05 by Mann-Whitney U test. Scale bar: 50 µm. (**B**) Immunofluorescent staining of macrophages by anti-F4/80 antibody in cystic kidneys of *Pkd1^flox/flox^*:*Pkhd1-Cre*:*CD74^+/+^* mice and *Pkd1^flox/flox^*:*Pkhd1-Cre*:*CD74^−/−^* mice. The average numbers of F4/80 positive cells per high power field image were present. Arrows indicate the F4/80 positive cells. Green: F4/80. Blue: DAPI. *n* = 3. * *p* ≤ 0.05 by Mann-Whitney U test. Scale bar: 50 µm. (**C**) The mRNA expression of MCP-1, TNF-α, and MIF in cystic kidneys of *Pkd1^flox/flox^*:*Ksp-Cre*:*CD74^+/+^* mice (*n* = 3) and *Pkd1^flox/flox^*:*Ksp-Cre*:*CD74^−/−^* mice (*n* = 3) analyzed by qRT-PCR. * *p* ≤ 0.05 by Mann-Whitney U test. (**D**) The mRNA expression of MCP-1, TNF-α, and MIF in cystic kidneys of *Pkd1^flox/flox^*:*Pkhd1-Cre*:*CD74^+/+^* mice (*n* = 3) and *Pkd1^flox/flox^*:*Pkhd1-Cre*:*CD74^−/−^* mice (*n* = 3) analyzed by qRT-PCR. * *p* ≤ 0.05 by Mann-Whitney U test.

We and others showed that macrophage promotes renal cyst formation in several ADPKD animal models [6,7,16]. We found that knockout of CD74 significantly decreased the recruitment of F4/80 positive macrophages in the pericystic site and interstitium of *Pkd1^flox/flox^*:*Ksp-Cre*:*CD74^−/−^* (Figure 4A) and *Pkd1^flox/flox^*:*Pkhd1-Cre*:*CD74^−/−^* kidneys (Figure 4B). It has been reported that the expression of inflammatory factors, including monocyte chemoattractant protein 1 (MCP-1), tumor necrosis factor alpha (TNF-α), and MIF, were increased in mouse and human ADPKD kidneys, and contribute to cystogenesis through regulating multiple signaling pathways [5,16,22]. We further found that knockout of CD74 significantly decreased the expression of MCP-1, TNF-α, and MIF in kidneys from *Pkd1^flox/flox^*:*Ksp-Cre*:*CD74^−/−^* and *Pkd1^flox/flox^*:*Pkhd1-Cre*:*CD74^−/−^* mice (Figure 4C,D). Our results suggested that CD74 may be through regulating the expression of inflammatory factors to promote macrophage accumulation in cystic kidneys.

### 3.4. Upregulation of CD74 Promotes Renal Fibrosis in Pkd1^flox/flox^:Pkhd1-Cre Mice

It has been reported that pro-inflammatory factors and pro-fibrotic growth factors secreted by inflammatory cells not only trigger renal epithelial cell proliferation but also the activation of myofibroblasts, which leads to interstitial fibrosis and end stage kidney disease (ESKD) in ADPKD [23]. The proteins of extracellular matrix (ECM) genes, such as collagen types I and III (Col I and Col III), fibronectin and α-SMA, are highly expressed by activated myofibroblasts and deposited in the interstitium [24]. We found that the mRNA levels of transforming growth factor β (TGF-β), α-SMA, Col I and Col III were increased in cystic kidneys from PN21 and PN28 *Pkd1^flox/flox^*:*Pkhd1-Cre* mice versus those in kidneys from age matched wild type mice as examined by qRT-PCR analysis (Supplemental Figure S2). Knockout of CD74 decreased the expression of Col I and Col III mRNAs in *Pkd1^flox/flox^*:*Pkhd1-Cre* mouse kidneys, but not the mRNA levels of TGF-β and Fibronectin (Supplemental Figure S3A). The deposition of α-SMA was decreased in kidneys of *Pkd1^flox/flox^*:*Pkhd1-Cre*:*CD74^−/−^* mouse versus *Pkd1^flox/flox^*:*Pkhd1-Cre*:*CD74^+/+^* mice as examined by immunohistochemistry (IHC) staining (Supplemental Figure S3B). As a consequence, deletion of CD74 decreased interstitial fibrosis in kidneys of *Pkd1^flox/flox^*:*Pkhd1-Cre* mice as examined by Masson Trichrome staining (Supplemental Figure S3C).

### 3.5. Targeting MIF with Its Inhibitor ISO-1 Decrease Renal Fibrosis in Pkd1 Hypomorphic Mouse Kidneys

**Figure 5:**
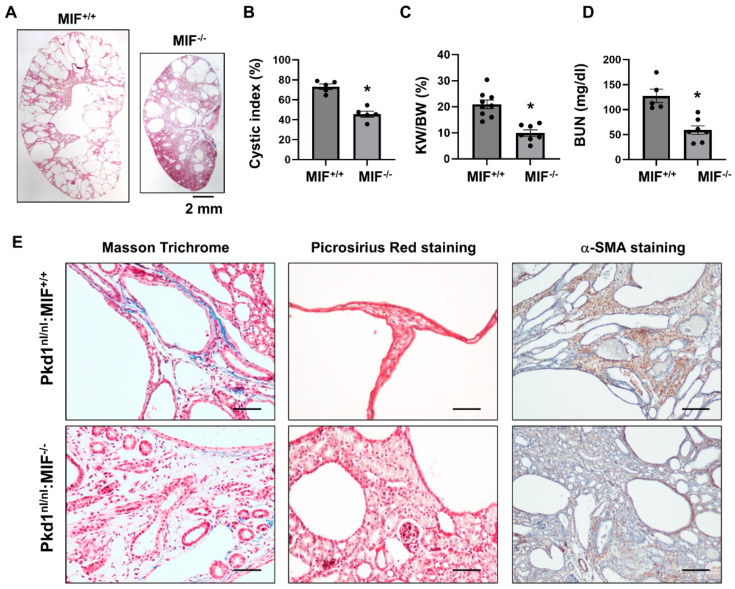
Knockout of MIF in *Pkd1^nl/nl^* mice delayed cyst formation and ameliorated the interstitial fibrosis. (**A**) Histologic examination of PN28 kidneys from *Pkd1^nl/nl^*:*MIF^+/+^* (MIF*^+/+^*) mice (*n* = 9) and *Pkd1^nl/nl^*:*MIF^−/−^* (MIF*^−/−^*) mice (*n* =7). Scale bar: 2 mm. (**B**) Cystic index was significantly decreased in PN28 kidneys from *MIF^−/−^* mice compared to *MIF^+/+^* mice. Mean ± SEM of all sections quantified for each condition are shown. (**C**,**D**) KW/BW ratios (**C**) and BUN levels (**D**) of PN28 *CD74^+/+^* and *CD74^−/−^* mice. Mean ± SEM. * *p* ≤ 0.05 is shown by student’s *t*-test. (**E**) The Masson Trichrome (blue, collagen), Picrosirius Red (Red, collagen) and α-SMA (Brown, α-SMA) staining of kidneys from PN28 *Pkd1^nl/nl^*:*MIF^+/+^* (MIF*^+/+^*) and *Pkd1^nl/nl^*:*MIF^−/−^* (MIF*^−/−^*) mice.

**Figure 6:**
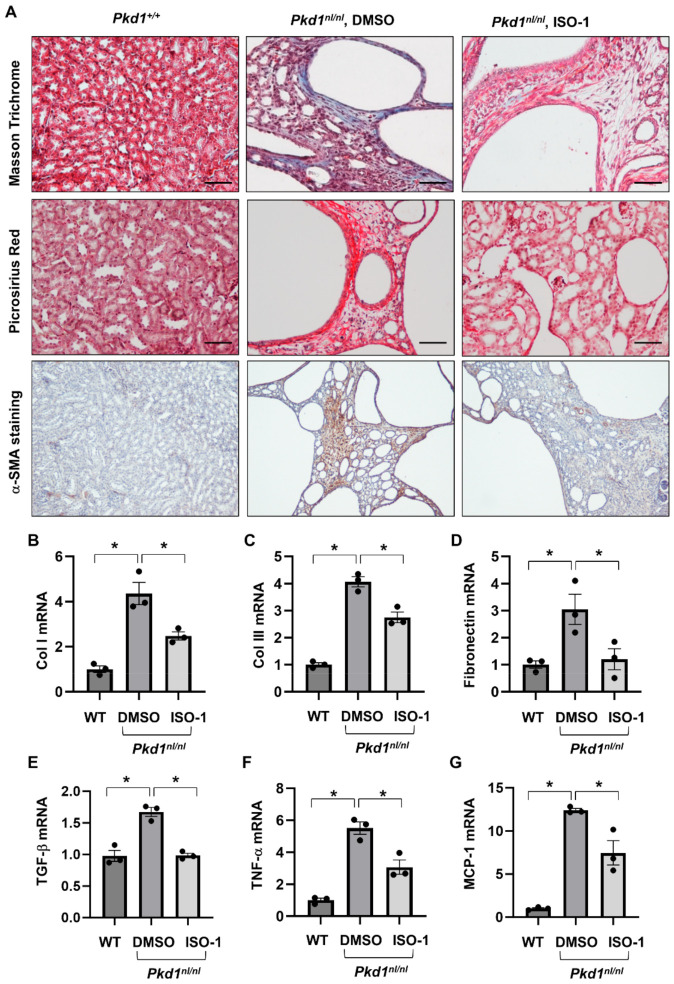
Treatment with ISO-1 ameliorated the interstitial fibrosis in *Pkd1^nl/nl^* mice. (**A**) The Masson Trichrome (top panel, blue, collagen), Picrosirius Red (middle panel, Red, collagen) and α-SMA (bottom panel, Brown, α-SMA) staining of kidneys from PN28 *Pkd1^+/+^* mice, *Pkd1^nl/nl^* mice treated with vehicle control, and *Pkd1^nl/nl^* mice treated with ISO-1. (**B**–**G**) The mRNA expression of Col I (**B**), Col III (**C**), Fibronectin (**D**), TGF-β (**E**), TNF-α (**F**) and MCP-1 (**G**) in kidneys from PN28 *Pkd1 ^+/+^* mice, *Pkd1^nl/nl^* mice treated with vehicle control, and *Pkd1^nl/nl^* mice treated with ISO-1 as analyzed by qRT-PCR; * *p* ≤ 0.05 by Mann-Whitney U test.

We have reported that knockout of MIF and targeting MIF with its inhibitor, ISO-1 delayed cyst growth in *Pkd1* knockout mouse models [16]. However, whether targeting MIF also decreases renal fibrosis in *Pkd1* mutant kidneys remains unknown. As CD74 is a major receptor of MIF, we hypothesize that targeting MIF should also decrease renal fibrosis in ADPKD kidneys. The *Pkd1* hypomorphic *Pkd1^nl/nl^* mouse has been reported to develop interstitial fibrosis during cyst progression [25]. First, we confirmed that the mRNA expression of TGF-β, α-SMA, Col I, Col III and fibronectin was strikingly upregulated in kidneys of PN28 *Pkd1^nl/nl^* mice compared to those of age matched wild type mice (Supplemental Figure S4A), and interstitial fibrosis was increased in kidneys of PN28 *Pkd1^nl/nl^* mice as detected by Masson Trichrome staining (Supplemental Figure S4B) and Picrosirus Red staining (Supplemental Figure S4C). To support our hypothesis, we generated *Pkd1^nl/nl^*:*MIF^−/−^* mice and found that knockout of MIF not only slowed cyst growth as characterized by decreased cyst index, KW/BW ratios, and BUN levels (Figure 5A–D), but also decreased interstitial fibrosis and α-SMA deposition (Figure 5E), as well as the mRNA expression of TGF-β, Col I and Col III in kidneys of PN28 *Pkd1^nl/nl^*:*MIF^−/−^* mice versus *Pkd1^nl/nl^*:*MIF^+/+^* mice (Supplemental Figure S5). We further found that treatment with an MIF inhibitor, ISO-1, decreased interstitial fibrosis as examined by Masson Trichrome staining and Picrosirus Red staining (Figure 6A) and the accumulation of α-SMA (Figure 6A, bottom panels) in kidneys from PN28 *Pkd1^nl/nl^* mice. Treatment with ISO-1 also normalized the expression of the upregulated fibrotic markers, including Col I, Col III, and fibronectin in *Pkd1^nl/nl^* mice (Figure 6B–D). In addition, the mRNA levels of TGF-β, TNF-α and MCP-1, which were upregulated in kidneys of *Pkd1^nl/nl^* mice versus age matched wild type mice, were decreased by ISO-1 treatment (Figure 6E–G). Our results suggested that MIF may be through its receptor CD74 to promote interstitial fibrosis in ADPKD kidneys, and targeting MIF with ISO-1 should ameliorate not only cyst growth but also interstitial fibrosis.

### 3.6. MIF-CD74 Signaling Activates Renal Fibroblasts

To investigate how MIF regulates renal fibrosis through CD74, we treated rat renal fibroblast NRK-49F cells with either MIF or ISO-1. We found that MIF stimulation promoted the proliferation of NRK-49F cells as examined by MTS assay (Supplemental Figure S6A), and induced the expression of proliferative marker PCNA (Supplemental Figure S6B). Similar with that of renal epithelial cells, MIF stimulation also increased the phosphorylation of ERK, AKT, and S6 in NRK-49F cells (Supplemental Figure S6C), which suggested that MIF regulated fibroblast cell proliferation by activating ERK, Akt and mTOR pathways. Knockdown of CD74 with siRNA blocked MIF induced activation and phosphorylation of ERK, mTOR, S6 and Rb in NRK-49F cells (Supplemental Figure S7A), suggesting that MIF regulated those signaling pathways through CD74.

**Figure 7:**
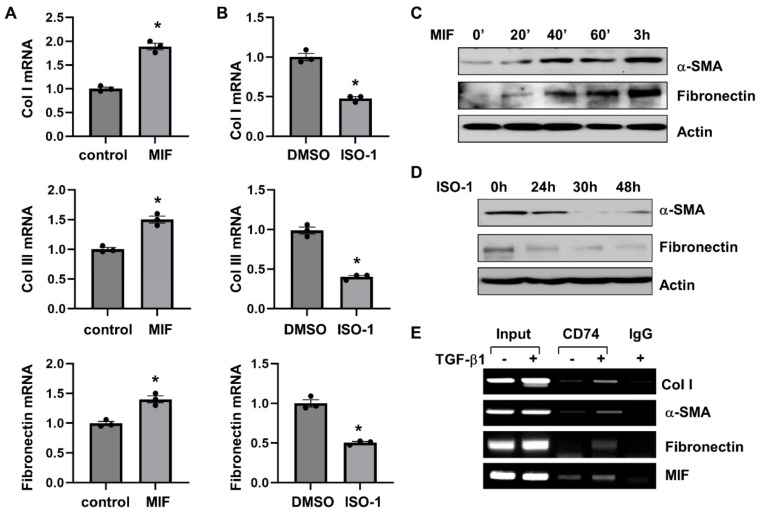
MIF-CD74 pathway regulates the expression of fibrotic markers. (**A**,**B**) The mRNA expression of Col I, Col lll and Fibronectin in NRK-49F cells stimulated with MIF or vehicle control (**A**), and NRK-49F cells treated with ISO-1 or vehicle control (**B**) *n* = 3. * *p* ≤ 0.05 by Mann-Whitney U test. (**C**,**D**) Western blot analysis of the expression of α-SMA and fibronectin in NRK-49F cells stimulated with MIF or vehicle control (**C**), and NRK-49F cells treated with ISO-1 or vehicle control (**D**). (**E**) The binding of CD74 on the promoters of Col I, α-SMA, fibronectin, and MIF in NRK-49F cells stimulated with TGF-β or vehicle control for 24 h analyzed by ChIP-PCR.

We further found that MIF stimulation increased the mRNA expression of fibrotic markers, including Col I, Col III and fibronectin, as analyzed by qRT-PCR (Figure 7A), and the protein expression of α-SMA and fibronectin as analyzed by Western blot (Figure 7C) in NRK-49F cells. Treatment with ISO-1 decreased the mRNA expression of Col I, Col III and fibronectin (Figure 7B), and the protein expression of α-SMA and fibronectin (Figure 7D) in NRK-49F cells. Knockdown of CD74 with siRNA decreased the mRNA levels of fibrotic markers, including Col I, Col III, α-SMA, and fibronectin, in NRK-49F cells with or without the treatment of MIF (Supplemental Figure S7B). To further understand the mechanisms of how CD74 regulates the expression of fibrotic markers, we found that CD74 bound to the promoters of Col I and α-SMA, but not to the promoter of fibronectin in NRK-49F cells without TGF-β treatment as examined with ChIP assay (Figure 7E). After treatment with TGF-β, CD74 bound to the promoter of fibronectin, and the binding of CD74 to the promoters of Col I and α-SMA was increased (Figure 7E). In addition, we found that CD74 bound to the promoter of MIF in NRK-49F cells with or without the stimulation of TGF-β (Figure 7E). Furthermore, treatment with TGF-β increases the entry of CD74 to nucleus compared to vehicle treated controls as examined by confocal macroscopy (Supplemental Figure S8). These results suggests that CD74 can function as a transcriptional factor to regulate gene expression in ADPKD kidneys.

### 3.7. Targeting of MIF-CD74 Signaling with ISO-1 Blocked the Activation of Renal Fibroblasts Induced by TGF-β

**Figure 8:**
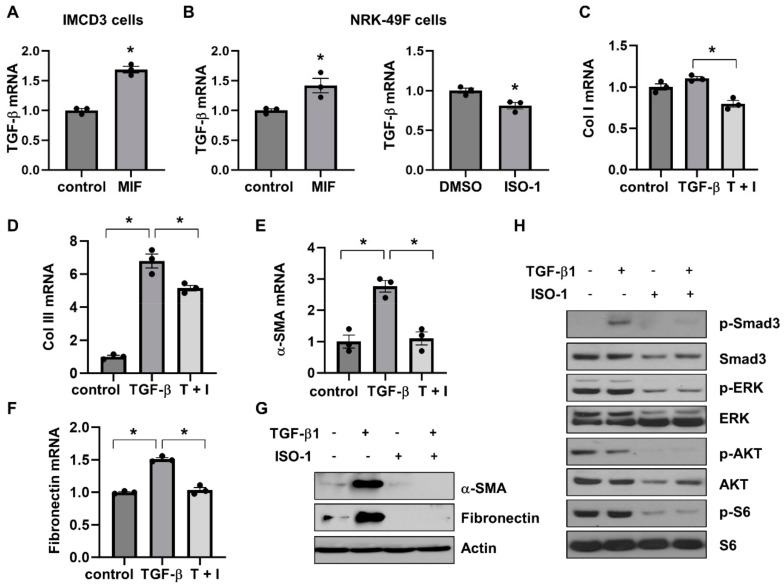
The association of MIF/CD74 and TGF-β/Smad3 pathways. (**A**) The mRNA expression of TGF-β in IMCD3 cells stimulated with MIF or vehicle control. (**B**) The mRNA expression of TGF-β in NRK-49F cells stimulated with MIF or vehicle control, and NRK-49F cells treated with ISO-1 or vehicle control. *n* = 3. * *p* ≤ 0.05 by Mann-Whitney U test. (**C**–**F**) The mRNA expression of Col I (**C**), Col lll (**D**), α-SMA (**E**), and fibronectin (**F**) in NRK-49F cells treated with TGF-β, TGF-β plus ISO-1, or vehicle control. *n* = 3. * *p* ≤ 0.05 by one-way ANOVA test. (**G**) Western blot analysis of the expression of α-SMA and fibronectin in NRK-49F cells treated with TGF-β, ISO-1, TGF-β plus ISO-1, or vehicle control for 48 h. (**H**) Western blot analysis of the expression of phospho-Smad3, Smad3, phospho-ERK, ERK, phospho-AKT, AKT, phospho-S6 and S6 in NRK-49F cells treated with TGF-β, ISO-1, TGF-β plus ISO-1, or vehicle control for 30 min.

Activation of TGF-β-SMAD signaling pathways plays a crucial role in promoting tissue fibrosis. Our results found that both MIF-CD74 and TGF-β-Smad pathways were activated in cystic kidneys [16,17]. We further investigated the association of MIF-CD74 and TGF-β-SMAD pathways in renal epithelial cells and fibroblasts. Our findings were as follows: (1) stimulation with MIF increased the mRNA expression of TGF-β in IMCD3 cells (Figure 8A) and NRK-49F cells (Figure 8B, left panel); (2) treatment with ISO-1 decreased the mRNA expression of TGF-β in NRK-49F cells (Figure 8B, right panel); (3) treatment with ISO-1 decreased the mRNA levels of fibrotic markers, including Col I, Col III, α-SMA, and fibronectin in NRK-49F cells induced by TGF-β (Figure 8C–F); and (4) treatment with ISO-1 also decreased the protein levels of α-SMA and fibronectin in NRK-49F cells induced by TGF-β (Figure 8G). In addition, treatment with TGF-β induced the phosphorylation of Smad3 in NRF-49F cells, whereas cotreatment with TGF-β and ISO-1 decreased the level of phospho-Smad3 (Figure 8H). We further found that treatment with TGF-β did not affect the phosphorylation of ERK, AKT, and S6 in NRK-49F cells that was induced by MIF and inhibited by ISO-1 (Supplemental Figure S6C and Figure 8H). These results suggested that MIF-CD74 signaling should regulate renal fibrosis in ADPKD kidneys through promoting the expression of TGF-β and the activation of Smad3, and targeting MIF with ISO-1 ameliorated interstitial fibrosis by blocking TGF-β induced activation of SMAD3.

## 4. Discussion

**Figure 9:**
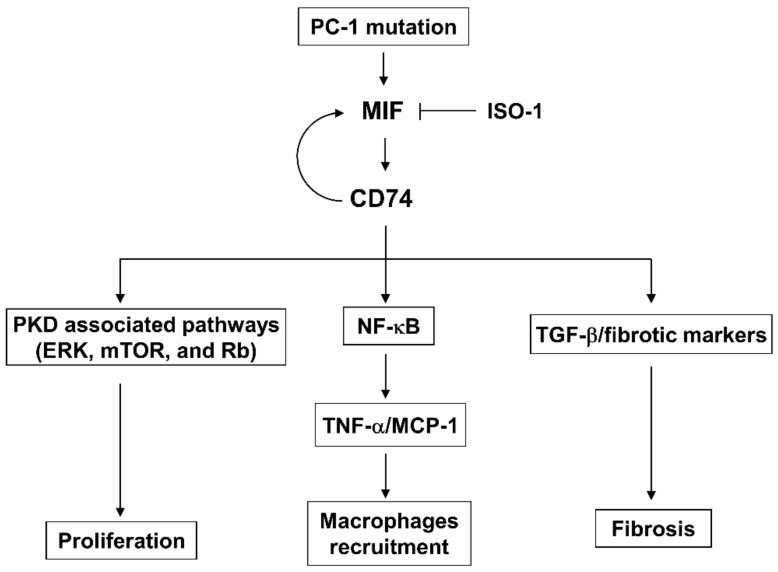
A schematic diagram depicting CD74-medicated pathways in ADPKD. CD74 regulates (1) cystic renal epithelial cell proliferation by activation of PKD associated pathways, including ERK, mTOR, and Rb, (2) the activation of NF-κB to increase the expression of TNF-α and MCP-1, leading to the accumulation of macrophages in ADPKD kidneys, (3) the transcription of fibrotic marker genes to regulate renal fibrosis, and (4) the transcription of MIF to form a positive feedback loop, to promote cyst growth and renal fibrosis in ADPKD.

In this study, we investigate the roles and mechanisms of upregulation of CD74 in promoting cyst growth and renal fibrosis in ADPKD. We show that knockout of CD74 ameliorates cyst growth and renal fibrosis in *Pkd1* mutant mouse kidneys. CD74 regulates renal epithelial cell proliferation mediated by PKD associated signaling pathways, including ERK, mTOR and Rb, and the activation of NF-κB. It also regulates the expression of MCP-1 and TNF-α and increases the recruitment of macrophages. CD74 regulates the transcription of fibrotic genes, including Col I, fibronectin and α-SMA through binding to their promoters, and the activation of TGF-β-Smad3 signaling to increase renal fibrosis in ADPKD kidneys (Figure 9). Knockout of *MIF* also decreases renal fibrosis in *Pkd1* mutant mouse kidneys, and knockdown of CD74 blocks MIF induced the expression of fibrotic markers, supporting a novel mechanism that MIF is through CD74 to regulate the transcription of fibrotic markers followed by renal fibrosis. Unexpectedly, CD74 also regulates the transcription of MIF by binding to its promoter, which forms a positive feedback loop between MIF and CD74, as MIF binds with CD74 to activate downstream signaling pathways and CD74 transcriptionally regulates the expression of MIF to facilitate this process.

As a receptor of MIF, CD74 regulates diverse signaling pathways including Akt, ERK, mTOR, Rb, Src and p53 in different cell types [26,27,28,29,30,31,32,33,34]. Notably, these pathways are hyperactive in PKD [2,3,5,19,35,36,37,38,39]. Our previous study indicated that MIF regulates renal epithelial cell proliferation through activation of ERK and mTOR signaling pathways. The levels of phospho-ERK and phospho-S6 were decreased in CD74 and *Pkd1* double knockout kidneys compared to that in *Pkd1* single knockout kidneys, supporting that knockout of CD74 delays cyst growth through normalization of these signaling pathways to regulate cystic renal epithelial cell proliferation and apoptosis. However, the molecular mechanisms linking MIF-CD74 complex with their downstream pathways are not fully explored yet.

It has been reported that MIF binds with CD74, leading to the release of the cytosolic intracellular domain of CD74 (CD74-ICD). CD74-ICD forms a complex with the p65 subunit of NF-κB and translocates into the nucleus to regulate the expression of NF-κB target genes, TRAF1 and BIRC3 in chronic lymphocytic B cells [40]. In normal B cells, CD74-ICD is translocated into the nucleus through an interaction with transcription factor PAX5 to regulate gene transcription [41]. It has been proposed that the putative transcriptional regulation induced by the CD74-ICD is indirect, which requires interacting proteins, such as the p65 subunit of NF-κB and the coactivating protein TAFII105 to mediate this effect [40,41]. However, it remained unclear whether CD74-ICD interacts directly with any of these two proteins or whether other, as yet unknown, interaction partners of the ICD are necessary to mediate these effects. We found that the activated form of p65, phospho-p65, was significantly decreased in CD74 and *Pkd1* double knockout kidneys compared to that in *Pkd1* single knockout kidneys (Figure 3D). We further show for the first time that CD74 can function as a transcriptional factor to directly regulate the transcription of fibrotic genes, including Col I, fibronectin and α-SMA, through binding to their promoters. Treatment with TGF-β increased the nuclear localization of CD74 and the binding of CD74 with the promoters of Col I, α-SMA, and fibronectin in NRK-49F cells. However, the transcriptional program triggered by CD74 should be fully defined so that, the CD74 ChIP-sequencing can be performed to identify additional targets of CD74 in the future.

The production of MIF is increased in both immune and non-immune mediated kidney diseases, which promotes inflammation and kidney injury by the accumulation of macrophages and T-cells [9,42]. Mice with MIF deletion are protected from immune-mediated lupus nephritis and treatment with a neutralizing anti-MIF antibody ameliorates kidney injury in crescentic anti-GBM glomerulonephritis [43,44]. Consistent with the protein expression of MIF, the mRNA expression of MIF was also decreased in *Pkd1* and CD74 double knockout kidneys (Figure 4C). We show that CD74 also binds to the promoter of MIF to regulate its transcription. This result forwards our understanding about the relationship between MIF and CD74, in that MIF binds with its receptor CD74 to regulate the activity of intracellular signaling pathways and CD74 also regulates the transcription of MIF to form a positive feedback loop during cyst progression in ADPKD and, possibly, in other diseases. In addition, this study addresses an unknown mechanism of how the upregulation of CD74 promotes the expression of MIF in disease condition.

TGF-β is the most important regulator of renal fibrosis [45,46,47]. The expression of TGF-β is elevated and TGF-β mediated Smad signaling is activated in ADPKD kidneys, which leads to renal fibrosis and atrophy [25]. The mechanisms for the activation of TGF-β signaling in ADPKD are not yet fully understood. In this study, we show a novel mechanism of the regulation of TGF-β transcription by MIF-CD74 in renal epithelial cells and fibroblasts, which should contribute to the activation of Smad3 signaling to promote renal fibrosis in ADPKD kidneys. Our study suggests that MIF triggers the activation of TGF-β-Smad signaling in a CD74 dependent manner via CD74 mediated the transcription regulation of TGF-β and fibrotic marker genes to regulate renal fibrosis in ADPKD kidneys. CD74 acts as a transcriptional factor to regulate the mRNA expression of Col I, α-SMA, and fibronectin (Figure 7E). Knockout of CD74 or MIF also decreased the mRNA level of Col III in kidneys from *Pkd1^flox/flox^*:*Pkhd1-Cre* and *Pkd1^nl/nl^* mice, respectively (Supplemental Figures S3A and S5). However, we found that CD74 did not directly bind to the promoter of Col III in NRK-49F cells, suggesting that another mechanism is involved in the transcriptional regulation of Col III in *Pkd1* mutant kidneys. It has been reported that the transcription of Col III can be regulated by the TGF-β and mitogen-activated protein kinase (MAPK) pathway [48]. MIF treatment activated the MAPK pathway in NRK-49F cells (Supplemental Figure S6C). Therefore, MIF-CD74 might activate the transcription of Col III through MAPK pathway in renal fibroblasts.

In sum, this study elucidates the roles of CD74 as key regulators of cyst formation through, (1) activation of PKD associated pathways to regulate cystic renal epithelial cell proliferation, (2) activation of NF-κB to upregulate the expression of TNF-α and MCP-1, resulting in the accumulation of macrophages, (3) transcriptional regulation of the expression of fibrotic markers to regulate renal fibrosis, and (4) transcriptional regulation of the expression of MIF to form a positive feedback loop to regulate cyst growth and renal fibrosis (Figure 9). Our data suggests that targeting MIF-CD74 may be a therapeutic strategy for ADPKD treatment in clinical setting. To enhance the generalizability of our findings, further exploration on a larger scale is worthy. Thus, this study not only leads to a better understanding of the mechanism of renal cyst formation and interstitial fibrosis but also provides novel therapeutic potential for ADPKD.

## Data Availability

Data are contained within the article.

## Supplementary Materials

The following supporting information can be downloaded at: https://www.mdpi.com/article/10.3390/cells13060489/s1, Figure S1: Western blot analysis of the expression of CD74 in kidneys from 3 different *Pkd1^flox/flox^*:*Ksp-Cre*:*CD74^+/+^* mice and *Pkd1^flox/flox^*:*Ksp-Cre*:*CD74^−/−^* mice; Figure S2: The expression of fibrotic markers in kidneys of *Pkd1^flox/flox^*:*Pkhd1-Cre* mice; Figure S3: The interstitial fibrosis was decreased in kidneys of PKD1 and CD74 double knockout mouse; Figure S4: The interstitial fibrosis of *Pkd1^nl/nl^* mice; Figure S5: The mRNA expression of TGF-β, Col I and Col lll in cystic kidneys from PN28 *Pkd1^nl/nl^*:*MIF^+/+^* (MIF*^+/+^*) and *Pkd1^nl/nl^*:*MIF^−/−^* (MIF*^−/−^*) mice; Figure S6: MIF activates renal fibroblasts; Figure S7: The activation of renal fibroblasts by MIF is mediated by CD74; Figure S8: The localization of CD74 in NRK-49F cells treated with TGF-β and vehicle.
