# Supplementary material for: CD74 Promotes Cyst Growth and Renal Fibrosis in Autosomal Dominant Polycystic Kidney Disease
Source: Cells. 2024 Mar 11;13(6):489. doi: 10.3390/cells13060489 (PMC10968819; doi:10.3390/cells13060489)
Supplement: Supplementary file 1 [file cells-13-00489-s001.zip › cells-2837209-supplementary.pdf]

Supplemental figure legends

Supplemental Figure S1. Western blot analysis of the expression of CD74 in kidneys from 3 different *Pkd1*$^{flox/flox}$*:Ksp-Cre:CD74*$^{+/+}$ mice and *Pkd1*$^{flox/flox}$*:Ksp-Cre:CD74*$^{-/-}$ mice.

Supplemental Figure S2. The expression of fibrotic markers in kidneys of *Pkd1*$^{flox/flox}$*:Pkhd1-Cre* mice. (**A**-**B**) The mRNA expression of TGF-β, α-SMA, Col I and Col III in kidneys from PN21 (**A**) and PN28 (**B**) *Pkd1*$^{flox/flox}$*:Pkhd1-Cre* (Flox) mice and *Pkd1*$^{+/+}$*:Pkhd1-Cre* (WT) mice. n = 3. * $p \le 0.05$ by Mann-Whitney U test.

Supplemental Figure S3. The interstitial fibrosis was decreased in kidneys of PKD1 and CD74 double knockout mouse. (**A**) The mRNA expression of Col I, Col III, fibronectin and TGF-β in cystic kidneys of *Pkd1*$^{flox/flox}$*:Pkhd1-Cre:CD74*$^{+/+}$ mice and *Pkd1*$^{flox/flox}$*:Pkhd1-Cre:CD74*$^{-/-}$ mice as analyzed by qRT-PCR. n = 3. * $p \le 0.05$ by Mann-Whitney U test. NS, not significant. (**B**) The Immunohistochemistry staining of α-SMA in cystic kidneys of *Pkd1*$^{flox/flox}$*:Pkhd1-Cre:CD74*$^{+/+}$ mice and *Pkd1*$^{flox/flox}$*:Pkhd1-Cre:CD74*$^{-/-}$ mice. Brown: α-SMA. Scale bar: 50 µm. (**C**) The Masson Trichrome staining of cystic kidneys from *Pkd1*$^{flox/flox}$*:Pkhd1-Cre:CD74*$^{+/+}$ mice and *Pkd1*$^{flox/flox}$*:Pkhd1-Cre:CD74*$^{-/-}$ mice. Red: cytosol, Black: Nuclei, Blue: Collagen. Scale bar: 50 µm.

Supplemental Figure S4. The interstitial fibrosis of *Pkd1*$^{nl/nl}$ mice. (**A**) The mRNA expression of TGF-β, α-SMA, Col I and Col III and Fibronectin in kidneys of PN28 *Pkd1*$^{nl/nl}$ mice and *Pkd1* $^{+/+}$ mice. n = 3. * $p \le 0.05$ by Mann-Whitney U test. (**B**-**C**) The Masson Trichrome (**B**) and α-SMA (**C**) staining of kidneys from PN28 *Pkd1*$^{nl/nl}$ mice and *Pkd1* $^{+/+}$ mice. Scale bar: 50 µm.

Supplemental Figure S5. The mRNA expression of TGF-β, Col I and Col III in cystic kidneys of from PN28 *Pkd1*$^{nl/nl}$*:MIF*$^{+/+}$ (MIF$^{+/+}$) and *Pkd1*$^{nl/nl}$*:MIF*$^{-/-}$ (MIF$^{-/-}$) mice. n = 3. * $p \le 0.05$ by Mann-Whitney U test.

Supplemental Figure S6. MIF activates renal fibroblasts. (**A**) The cell viability of NRK-49F cells treated with MIF at indicated concentrations by MTS assay. (**B**) Western blot analysis of the expression of PCNA in NRK-49F cells treated with MIF at indicated time period. (**C**) Western blot analysis of the expression of phospho-ERK, ERK, phospho-AKT, AKT, phospho-S6 and S6 in NRK-49F cells treated with MIF at indicated time period.

Supplemental Figure S7. The activation of renal fibroblasts by MIF is mediated by CD74. (**A**) NRK-49F cells were transfected with control or CD74 siRNA for 48 hours, followed by MIF (10 ng/ml) for 2 hours. Cells were collected for Western blot analysis of the expression of phosph-ERK, ERK, phospho-mTOR, phospho-S6, S6, phospho-Rb, Rb, and CD74. (**B**) NRK-49F cells were transfected with control or CD74 siRNA for 48 hours, followed by MIF (10 ng/ml) for 2 hours. The mRNA expression of Col I, Col III, α-SMA, and fibronectin in these cells were analyzed by qRT-PCR. n = 3. * $p \le 0.05$ by one-way ANOVA test.

Supplemental Figure S8. The localization of CD74 in NRK-49F cells treated with TGF-β and vehicle. The immunostaining with CD74 antibody shows that treatment with TGF-β increases the entry of CD74 to nucleus compared to vehicle treated controls as examined by confocal macroscopy. *Green*: CD74, *Blue*: DAPI. Scale bar: 10 µm.

Supplemental Figure 1

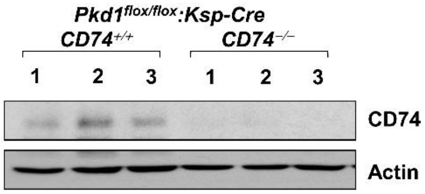

Supplemental Figure S1. Western blot analysis of the expression of CD74 in kidneys from 3 different *Pkd1*$^{flox/flox}$*:Ksp-Cre:CD74*$^{+/+}$ mice and *Pkd1*$^{flox/flox}$*:Ksp-Cre:CD74*$^{-/-}$ mice.

Supplemental Figure 2

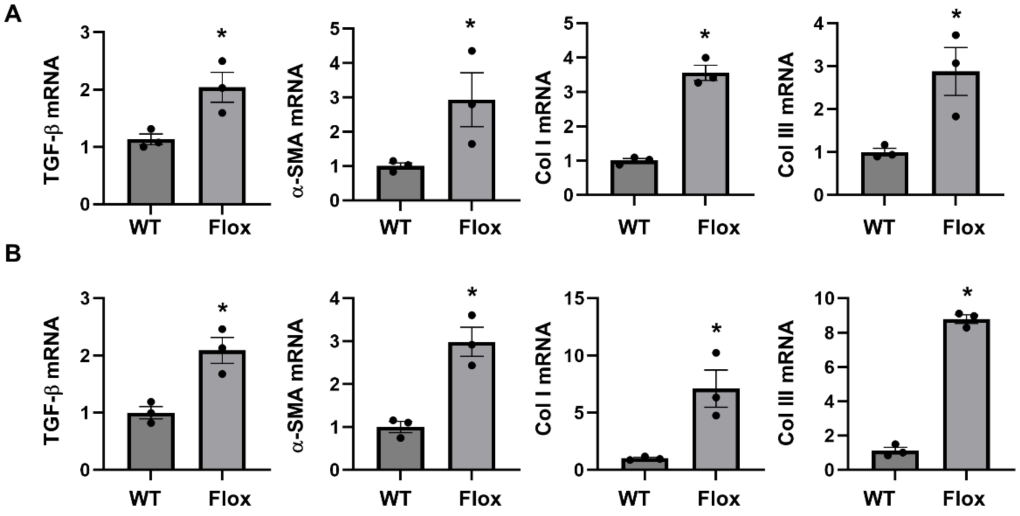

Supplemental Figure S2. The expression of fibrotic markers in kidneys of *Pkd1$^{flox/flox}$:Pkhd1-Cre* mice. (**A**-**B**) The mRNA expression of TGF-β, α-SMA, Col I and Col III in kidneys from PN21 (**A**) and PN28 (**B**) *Pkd1$^{flox/flox}$:Pkhd1-Cre* (Flox) mice and *Pkd1$^{+/+}$:Pkhd1-Cre* (WT) mice. n = 3. * $p \leq 0.05$ by Mann-Whitney U test.

Supplemental Figure 3

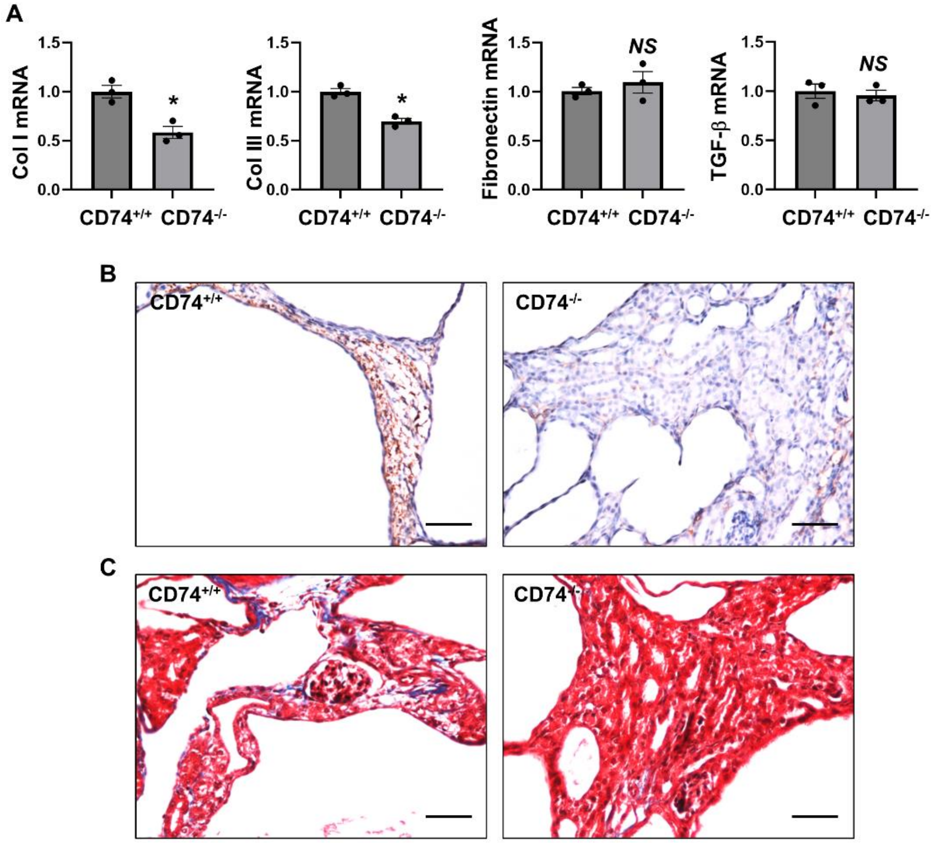

Supplemental Figure S3. The interstitial fibrosis was decreased in kidneys of PKD1 and CD74 double knockout mouse. (**A**) The mRNA expression of Col I, Col III, fibronectin and TGF-β in cystic kidneys of *Pkd1*$^{flox/flox}$*:Pkhd1-Cre:CD74*$^{+/+}$ mice and *Pkd1*$^{flox/flox}$*:Pkhd1-Cre:CD74*$^{-/-}$ mice as analyzed by qRT-PCR. n = 3. * $p \leq 0.05$ by Mann-Whitney U test. NS, not significant. (**B**) The Immunohistochemistry staining of α-SMA in cystic kidneys of *Pkd1*$^{flox/flox}$*:Pkhd1-Cre:CD74*$^{+/+}$ mice and *Pkd1*$^{flox/flox}$*:Pkhd1-Cre:CD74*$^{-/-}$ mice. Brown: α-SMA. Scale bar: 50 µm. (**C**) The Masson Trichrome staining of cystic kidneys from *Pkd1*$^{flox/flox}$*:Pkhd1-Cre:CD74*$^{+/+}$ mice and *Pkd1*$^{flox/flox}$*:Pkhd1-Cre:CD74*$^{-/-}$ mice. Red: cytosol, Black: Nuclei, Blue: Collagen. Scale bar: 50 µm.

Supplemental Figure 4

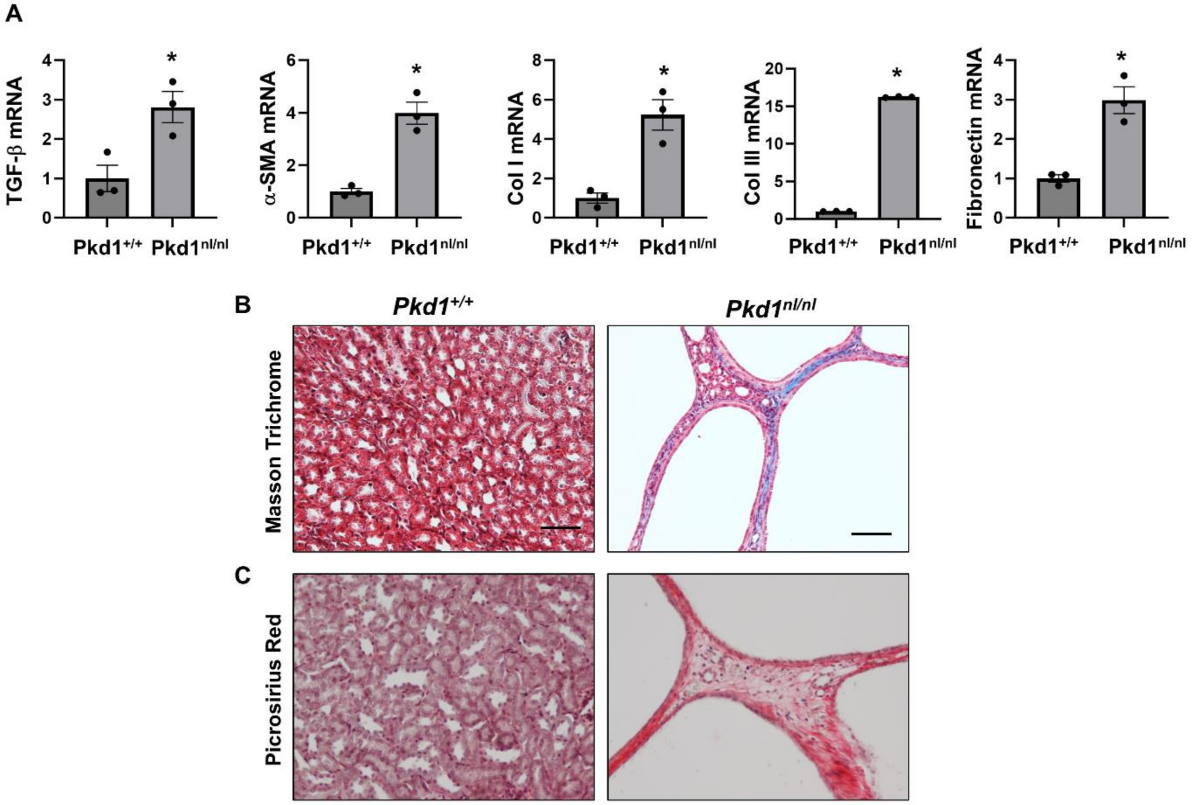

Supplemental Figure S4. The interstitial fibrosis of *Pkd1*$^{nl/nl}$ mice. (**A**) The mRNA expression of TGF-β, α-SMA, Col I and Col III and Fibronectin in kidneys of PN28 *Pkd1*$^{nl/nl}$ mice and *Pkd1* $^{+/+}$ mice. n = 3. * $p \leq 0.05$ by Mann-Whitney U test. (**B**-**C**) The Masson Trichrome (**B**) and α-SMA (**C**) staining of kidneys from PN28 *Pkd1*$^{nl/nl}$ mice and *Pkd1* $^{+/+}$ mice. Scale bar: 50 µm.

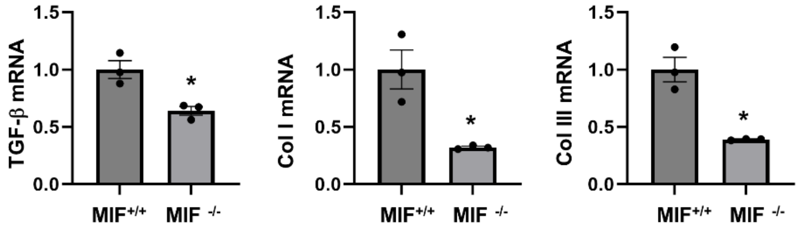

Supplemental Figure S5. The mRNA expression of TGF-β, Col I and Col lll in cystic kidneys from PN28 *Pkd1$^{nl/nl}$:MIF$^{+/+}$* (MIF$^{+/+}$) and *Pkd1$^{nl/nl}$:MIF$^{-/-}$* (MIF$^{-/-}$) mice. n = 3. * $p \leq 0.05$ by Mann-Whitney U test.

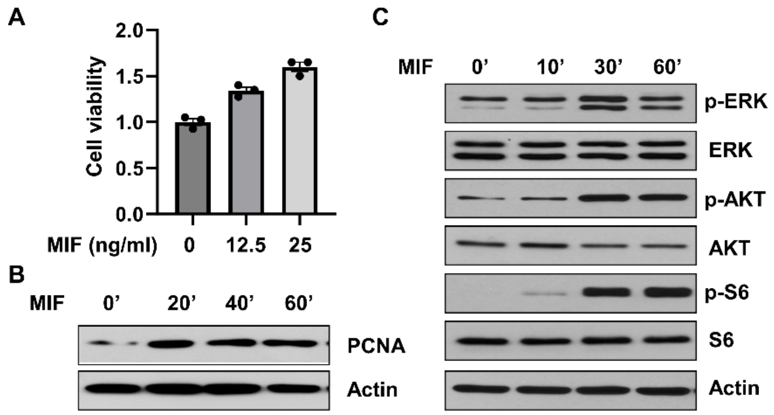

Supplemental Figure S6. MIF activates renal fibroblasts. (**A**) The cell viability of NRK-49F cells treated with MIF at indicated concentrations by MTS assay. (**B**) Western blot analysis of the expression of PCNA in NRK-49F cells treated with MIF at indicated time period. (**C**) Western blot analysis of the expression of phospho-ERK, ERK, phospho-AKT, AKT, phospho-S6 and S6 in NRK-49F cells treated with MIF at indicated time period.

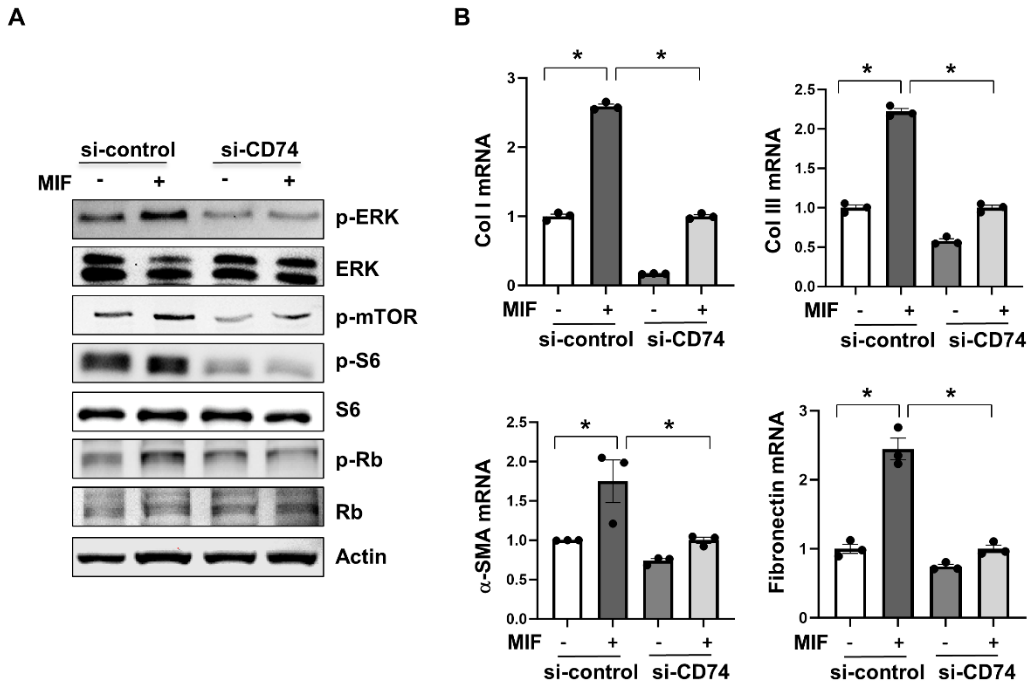

Supplemental Figure SS7. The activation of renal fibroblasts by MIF is mediated by CD74. (**A**) NRK-49F cells were transfected with control or CD74 siRNA for 48 hours, followed by MIF (10 ng/ml) for 2 hours. Cells were collected for Western blot analysis of the expression of phosph-ERK, ERK, phospho-mTOR, phospho-S6, S6, phospho-Rb, Rb, and CD74. (**B**) NRK-49F cells were transfected with control or CD74 siRNA for 48 hours, followed by MIF (10 ng/ml) for 2 hours. The mRNA expression of Col I, Col III, α-SMA, and fibronectin in these cells were analyzed by qRT-PCR. n = 3. * $p \leq 0.05$ by one-way ANOVA test.

Supplemental Figure 8

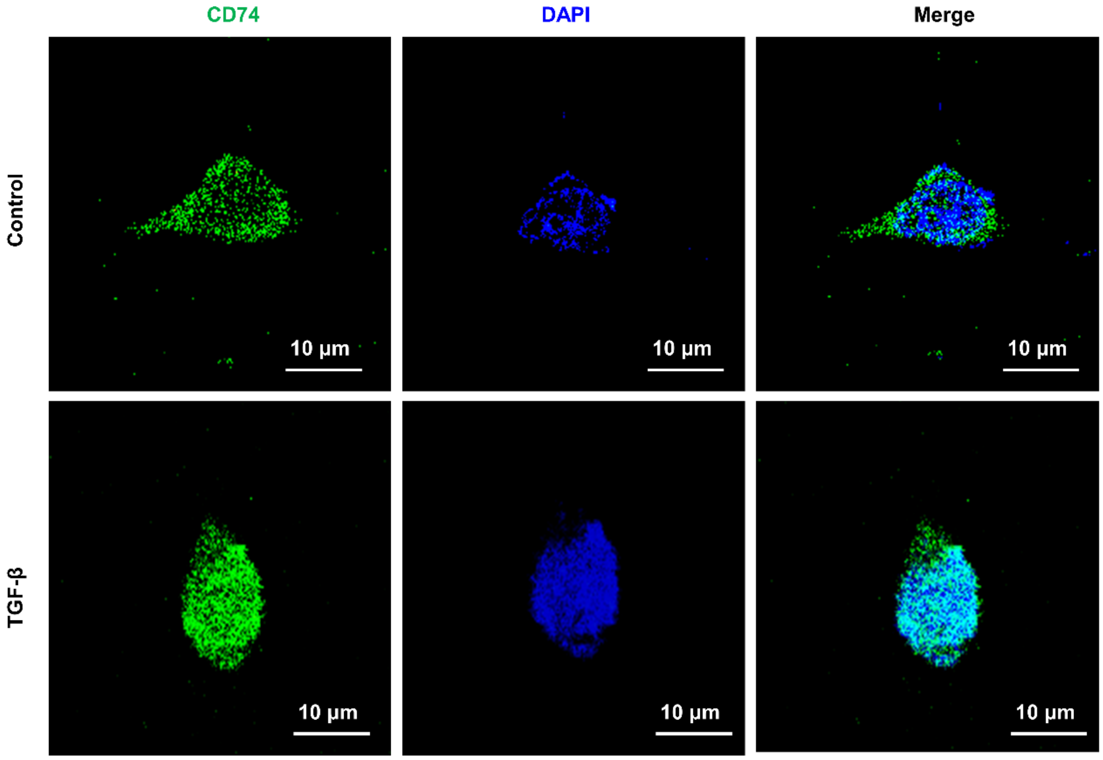

Supplemental Figure S8. The localization of CD74 in NRK-49F cells treated with TGF-β and vehicle. The immunostaining with CD74 antibody shows that treatment with TGF-β increases the entry of CD74 to nucleus compared to vehicle treated controls as examined by confocal macroscopy. *Green*: CD74, *Blue*: DAPI. Scale bar: 10 μm.
